# Supplementary material for: Establishment of Autoreactive CD4+CD8+ T Cell Hybridomas from Sjögren’s Disease Model, SATB1 Conditional Knockout Mice
Source: Int J Mol Sci. 2025 Dec 30;27(1):414. doi: 10.3390/ijms27010414 (PMC12787129; doi:10.3390/ijms27010414)
Supplement: Supplementary file 1 [file ijms-27-00414-s001.zip › ijms-4027518 supplement_rev.pdf]

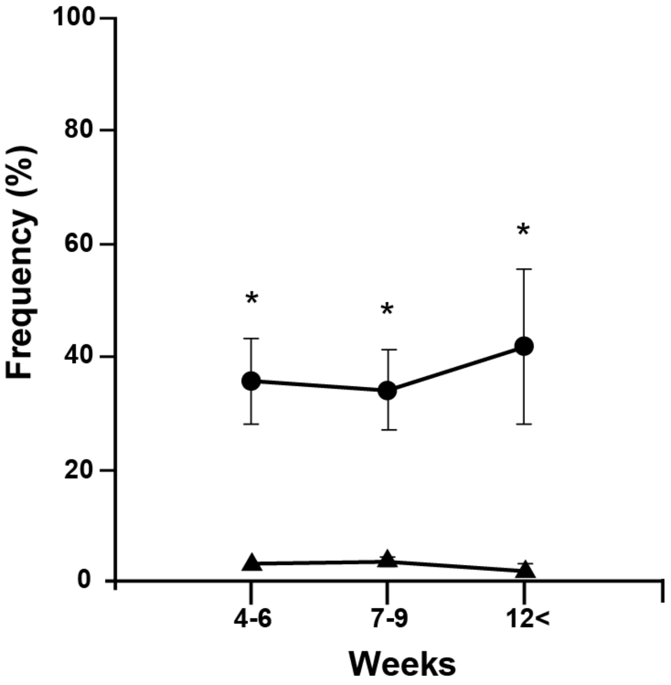

Figure S1. Frequency of DP T cells in the total T cell population in the spleen (triangles) and salivary glands (circles) of SATB1cKO mice at the different ages indicated. The data shown in Figure 1b were re-analyzed statistically by Student's t-test, although normality was not formally tested (*$p < 0.05$). Therefore, this figure is just indicative.

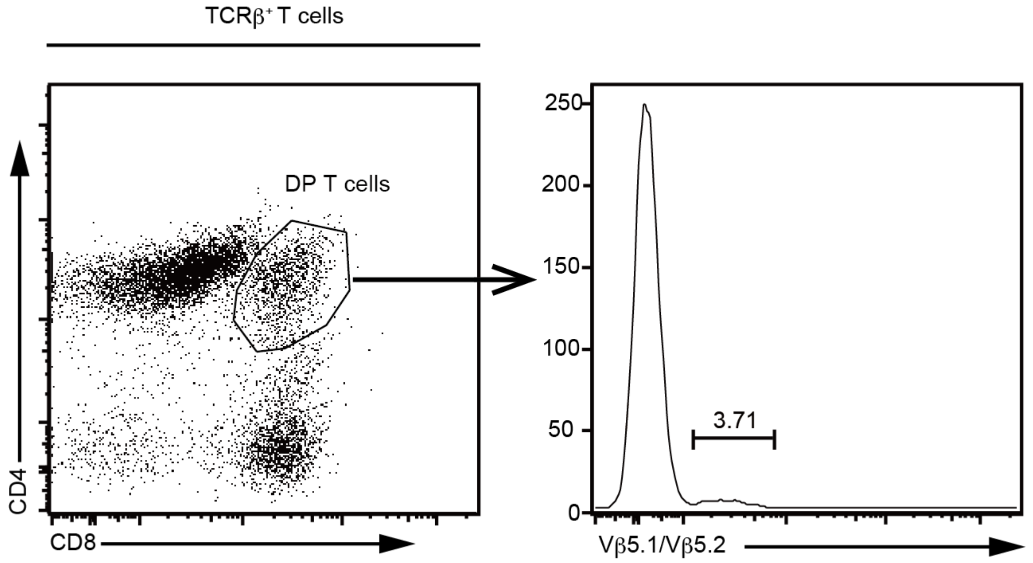

Figure S2. Vβ5.1 and Vβ5.2 expression in DP T cells from the cLNs of Rag2$^{-/-}$ mice engrafted with SATB1cKO cLN T cells. Twelve weeks after transfer, the cLNs of recipients were harvested and analyzed on FACS. Vβ5.1 and Vβ5.2 expression was examined in DP T cells within the TCRβ^{+} T cell population. One of representative plots is shown.

Table S1. The median value and interquartile range of the results shown in Figure 4.

Hybridoma	Treatment	Median	Interquartile range
G5-5B	-	0.996	0.981-0.998
	CD3/CD28	5.16	4.90-5.20
	Salivary gland	2.44	1.45-2.76
5	-	1.13	1.06-1.13
	CD3/CD28	191	184-199
	Salivary gland	245	195-294
35-10	-	1.00	0.983-1.13
	CD3/CD28	25.7	25.3-26.6
	Salivary gland	0.46	0.45-1.07
37-11	-	1.00	0.878-1.22
	CD3/CD28	129	129-133
	Salivary gland	77.1	71.1-91.3
C9-12-12	-	1.00	0.999-1.01
	CD3/CD28	23.7	23.1-23.8
	Salivary gland	18.2	16.1-19.6

Table S2. Forward primers for cDNA of murine TCRα and β chains

Primer name [*1]	Sequence (5´ to 3´) [*2]
mAL-1	TGGAGGAGAACCCTGGACCTatgctgcagatgtgggggtttg
mAL-2	TGGAGGAGAACCCTGGACCTatgaagcaggtggcaaaagtg
mAL-3-1	TGGAGGAGAACCCTGGACCTatgaagacRgtgactggacc
mAL-3-4	TGGAGGAGAACCCTGGACCTatgaaaacagtgRctggacc
mAL-4-2	TGGAGGAGAACCCTGGACCTatggagaggagcccgggaac
mAL-4-3	TGGAGGAGAACCCTGGACCTatgSagaggaacctgggagc
mAL-5-1	TGGAGGAGAACCCTGGACCTatgaagacagctattcatgc
mAL-5D-4	TGGAGGAGAACCCTGGACCTatgaaaacatatgctcctac
mAL-6-1	TGGAGGAGAACCCTGGACCTatgaactattctccagctttagtg
mAL-6-2	TGGAGGAGAACCCTGGACCTatgaacacttctccagctttag
mAL-6-3	TGGAGGAGAACCCTGGACCTatgaacaattccccagctttag
mAL-6-4	TGGAGGAGAACCCTGGACCTatgaatacttctccagttttag
mAL-6-5	TGGAGGAGAACCCTGGACCTatgaacctttgtcctgaactg
mAL-6-6	TGGAGGAGAACCCTGGACCTatggactYttctccaggcttc
mAL-7-1	TGGAGGAGAACCCTGGACCTatgaagtccttgtgtgtttcac
mAL-7-2	TGGAGGAGAACCCTGGACCTatgaaatcctttagtatttccctag
mAL-7-3	TGGAGGAGAACCCTGGACCTatgaaatccttgagtgtttc
mAL-7D-4	TGGAGGAGAACCCTGGACCTatgaaatccttgagtgttttac
mAL-7-6	TGGAGGAGAACCCTGGACCTatgcattccttacatgtttcac
mAL-8-1	TGGAGGAGAACCCTGGACCTatgcacagcctcctggggttg
mAL-8-2	TGGAGGAGAACCCTGGACCTatgaacagattcctgggaa
mAL-9-1	TGGAGGAGAACCCTGGACCTatgctcctggtYctcatctcg
mAL-9-2	TGGAGGAGAACCCTGGACCTatgctcctgYtgctcctcc
mAL-9D-4	TGGAGGAGAACCCTGGACCTatgctcctggcactcctcc
mAL-10	TGGAGGAGAACCCTGGACCTatgaagacatcccttcacactg
mAL-11*01	TGGAGGAGAACCCTGGACCTatgaaaaagtgccttagtgcctg
mAL-11*02	TGGAGGAGAACCCTGGACCTatgaaaaagYgcctgagtgcc
mAL-12-1	TGGAGGAGAACCCTGGACCTatgcgtcctgtcacctgctc
mAL-12D-1*01	TGGAGGAGAACCCTGGACCTatgaacatgcgtcctgWcac
mAL-12D-1*02	TGGAGGAGAACCCTGGACCTatgcgtcctgWcacctcctc
mAL-12-2	TGGAGGAGAACCCTGGACCTatgaacatgcatcctgtcacctg
mAL-12-3	TGGAGGAGAACCCTGGACCTatgcgtcctgRcacctgctca
mAL-13-1	TGGAGGAGAACCCTGGACCTatgaacaggctgctgtgctc
mAL-13D-1	TGGAGGAGAACCCTGGACCTatgaagaggctgctgagctc
mAL-13-2	TGGAGGAGAACCCTGGACCTatgaagaggctgatgtg
mAL-14-1	TGGAGGAGAACCCTGGACCTatggacaMgatcctgacagca
mAL-14D-1	TGGAGGAGAACCCTGGACCTatggacaagattctgacagcatc
mAL-14D-2	TGGAGGAGAACCCTGGACCTatggacaagaacctgacagca
mAL-15-1	TGGAGGAGAACCCTGGACCTatgcctcctcacagcctg
mAL-15-2	TGGAGGAGAACCCTGGACCTatgcctcctcagagcctgctc
mAL-16	TGGAGGAGAACCCTGGACCTatgctgattctaagcctg
mAL-16*01	TGGAGGAGAACCCTGGACCTatgaagaggctgctgtgc
mAL-16D	TGGAGGAGAACCCTGGACCTatgctcctggcactcctc
mAL-17	TGGAGGAGAACCCTGGACCTatgttccYagtgaccattctg
mAL-19	TGGAGGAGAACCCTGGACCTatgactggcttcctgaaggcc
mAL-21	TGGAGGAGAACCCTGGACCTatgggatgtgtgagtggaattgc

mBL-1	AAGGATCCGAATTCCTGCAGGatgtggcagttttgcattctgtg
mBL-2	AAGGATCCGAATTCCTGCAGGatgggctccattttcctcagttgc
mBL-3	AAGGATCCGAATTCCTGCAGGatggatatctggcttctaggttgg
mBL-4	AAGGATCCGAATTCCTGCAGGatgggctgtaggctcctaag
mBL-5	AAGGATCCGAATTCCTGCAGGatgagctgcaggcttctc
mBL-12-1	AAGGATCCGAATTCCTGCAGGatgtctaacactgtcctcgctg
mBL-12-2	AAGGATCCGAATTCCTGCAGGatgtctaacactgccttccctg
mBL-13-1	AAGGATCCGAATTCCTGCAGGatgggctccaggctctttc
mBL-13-2	AAGGATCCGAATTCCTGCAGGatgggctccaggctcttcttcg
mBL-13-3	AAGGATCCGAATTCCTGCAGGatgggctccagactcttctttg
mBL-14	AAGGATCCGAATTCCTGCAGGatgggcaccaggcttcttg
mBL-15	AAGGATCCGAATTCCTGCAGGatgggcatccagaccctctg
mBL-16	AAGGATCCGAATTCCTGCAGGatggcccccaggctccttttctg
mBL-17	AAGGATCCGAATTCCTGCAGGatggatcctagacttctttgctgtg
mBL-19	AAGGATCCGAATTCCTGCAGGatgaacaagtgggttttctgc
mBL-20	AAGGATCCGAATTCCTGCAGGatgttactgcttctattacttctgg
mBL-23	AAGGATCCGAATTCCTGCAGGatgggtgcacggctcatttg
mBL-24	AAGGATCCGAATTCCTGCAGGatgggtgcaagactgctctg
mBL-26	AAGGATCCGAATTCCTGCAGGatggctacaaggctcctctgt
mBL-29	AAGGATCCGAATTCCTGCAGGatgagagttaggctcatctctgc
mBL-30	AAGGATCCGAATTCCTGCAGGatgtggacattcctgctacttc
mBL-31	AAGGATCCGAATTCCTGCAGGatgctgtactctctccttgc

*1, mAL and mBL show primer for α and β chains, respectively. *2, Uppercases show adaptor sequece, used for 2nd PCR.
Lowercases show sequence specific for leader sequences of v

Table S3. Reverse primers used in a 1st PCR to amplify cDNA of murine TCRα and β chains

Primer name	Sequence (5´ to 3´)
Trac 3UTR PrR1	GAATCAGGGCCAACCAGACC
Trbc1 3UTR PrR1	TGTAGGCATTTTCCAGGTCACA
Trbc2 3UTR PrR1	CAAGGTGTCAACGAGGAAGGA

Table S4. Primers used in a 2nd PCR to amplify cDNA of murine TCRα and β chains

Primer name	Sequence (5´ to 3´)[*1]
P2A-C IF	GGATATCTGCAGAATtggaggagaaccctggacct
BES-AP IF	GGATATCTGCAGAATaaggatccgaattcctgcagga
Trac 3UTR IF R2	AGTAACGGCGCTAGCagggctcctttggcttgaag
Trbc1 3UTR IF R2	AGTAACGGCGCTAGCggcagaatttgggatgcacagacaaatgag
Trbc2 3UTR IF R2	AGTAACGGCGCTAGCaggaaaatctatggccaggg

*1, Uppercases is complementary to the end of cloning site of pCR vector.
Lowercases is spesific for 1st PCR fragments.

Table S5. Primers for DNA sequencing of pCR-TCRα or β clones

Primer name	Sequence (5´ to 3´)
M13 rev	CAGGAAACAGCTATGAC
T7pro	TAATACGACTCACTATAGG
